# Supplementary material for: T-Cell Assay after COVID-19 Vaccination Could Be a Useful Tool? A Pilot Study on Interferon-Gamma Release Assay in Healthcare Workers
Source: Diseases. 2022 Jul 31;10(3):49. doi: 10.3390/diseases10030049 (PMC9396988; doi:10.3390/diseases10030049)
Supplement: Supplementary file 1 [file diseases-10-00049-s001.zip › diseases-1768442-supplementary.pdf]

Supplemental material

Antibody monitoring	N° (Total 98)	N° of dropouts	N° of positive subjects at SARS-CoV-2 molecular swab during the period monitoring	N° of subjects in therapeutical care	N° of vaccinated subjects	T0: First dose	T1: Second dose	T2: 15 days after 2nd dose	T3: 45 days after 2nd dose	T4: 3 months after 2nd dose	T5: 6 months after 2nd dose	T6: 8 months after 2nd dose
						RBD median levels (BAU/mL)	RBD median levels (BAU/mL)	RBD median levels (BAU/mL)	RBD median levels (BAU/mL)	RBD median levels (BAU/mL)	RBD median levels (BAU/mL)	RBD median levels (BAU/mL)
**Group 1**	8	n/a	n/a	n/a	8	0.00 [IQR: 0.00 – 0.184]	63.56 [IQR: 60.52 – 70.70]	480.50 [IQR: 63.75 – 795.5]	454.50 [IQR: 305.00 – 530.25]	260.00 [IQR: 183.1 – 364.15]	52.23 [IQR: 43.84 – 66.00]	28.898 [IQR: 27.445 – 33.969]
**Group 2**	9	n/a	n/a	n/a	9	0.21 [IQR: 0.00 – 0.71]	141.25 [IQR: 24.40 – 298.23]	611.59 [IQR: 105.65 – 1291.33]	2648.18 [IQR: 457.46 – 5591.46]	1716.40 [IQR: 87.90 – 3392.50]	1485 [IQR: 1066.8 – 4330.00	645.295 [IQR: 376.343 – 1072.254]
**Group 3**	6	n/a	n/a	n/a	0	0.62 [IQR: 0.50 – 1.19]	0.12 [IQR: 0.10 – 0.71]	0.65 [IQR: 0.61 – 0.85]	1.17 [IQR: 0.69 – 3.20]	0.29 [IQR: 0.03 – 0.67]	1.41 [IQR: 0.75-2.00]	0.563 [IQR: 0.00 – 2.736]
**Subjects excluded**	75	8	10	1	56 subjects: 54 with fully complete dose + 2 subjects with one single dose booster	N=54 0.00 [IQR: 0.00 – 0.35]	N=54 171.92 [IQR: 63.86 – 363.12]	N=54 433.00 [IQR: 433.00 – 3363.11]	N=54 1346.63 [IQR: 311.63 – 2196.17]	N=54 430.00 [IQR: 321.00 – 746.40]	N=54 227.35 [IQR: 157.60 – 318.90]	N=54 125 [IQR: 62.52 – 299.99]

Table S1: Number of subjects and median value and interquartile range (IQR: 25th and 75th percentile) at all the times of samples collection of the period of monitoring [RBD: Receptor Binding Domain; BAU/mL: Binding Antibody Unit/ml; IQR: interquartile range; n/a: no applicable; T: time; N: number].
